# Spectacles or No Spectacles

**Published:** 1869-10

**Authors:** 


### Selections.

---

Spectacles or No Spectacles.—In No. 23, last volume (June 5), Dr. J. V. C. Smith advises the public to begin with the firm resolution never to wear glasses of any kind for reading or writing, but to attempt persistently to read without them, by which the eye will regain its former power. To strengthen his suggestion he brings names of celebrated persons who have done without them, still having the perfect use of their eyes to a good old age. Such an admonition is hardly necessary in this age of vanity, for it is usual now, that persons arriving at a period where the failing organs proclaim advancing age, strenuously resist the use of glasses, because they advertise the unwelcome fact.

The truth of the doctor's assertion consists in the fact that the eyes of some are probably susceptible to such a change; but it is only the empiric whose confidence is absolute and final, while the thinking professional makes experiments and watches the results of a trial.

It is easy to collect a small volume of telling examples to prove preposterous opinions, but that is no evidence. Experience must be our guide. Much depends on the individual case, much on the condition of the organism. What will help one won't help others; the great difficulty, beside, consists in deciding whether the beneficial effects attributed to any particular cause really has reference to its action or to some concurrent cause.

As it comes under our daily notice, the method recommended by the doctor has a directly opposite effect on the eye-sight, we can not withhold the suspicion that the recommendation put forth is a fallacy. Let the doctor make experiments, collect precise data; let him give us the maximum time during which we must grope in the dark in order to see light again.

Studious habits, overwork, the taxing of the eye to perform most severe duty for a considerable period of time, are the universal causes of the early failing of its functions, but the idea of relying upon time for its restoration, is utterly inadmissible, for if time is invoked at all, it must be invoked as the cause of the very evil which we thus propose to leave to its cure.

The progress of civilization, the art of printing, does a great deal toward the increase of weak sight, and as Gutenberg put forth his invention only in 1438, the ancients could not suffer from that source; but even before that date, in 1292, Roger Bacon mentions the benefit derived from the use of a plano-convex glass, by old men and those with weak eyes. This shows conclusively that although Cicero never complained of imperfect vision, even at the age of sixty-three (perhaps he had his, so-called, "second sight," an occurrence not very uncommon among aged people), there must have been many others who have suffered from that defect.

The Bible mentions that Isaac, the Patriarch, had dim eyes from old age.

Experience proves daily that the judicious use of glasses is mostly accompanied by beneficial results; therefore we should think, with due deference to Dr. Smith's opinion, that it is best to submit with good grace to an affliction which can not be averted.—*Scientific American.*

